# Passive and active parental food allocation in a songbird

**DOI:** 10.1093/beheco/arad043

**Published:** 2023-05-29

**Authors:** Daniel Parejo-Pulido, Lorenzo Pérez-Rodríguez, Inmaculada Abril-Colón, Jaime Potti, Tomás Redondo

**Affiliations:** Instituto de Investigación en Recursos Cinegéticos (IREC), CSIC-UCLM-JCCM, Ronda de Toledo 12, 13005 Ciudad Real, Spain; Instituto de Investigación en Recursos Cinegéticos (IREC), CSIC-UCLM-JCCM, Ronda de Toledo 12, 13005 Ciudad Real, Spain; Museo Nacional de Ciencias Naturales (MNCN), CSIC, Departamento de Ecología Evolutiva, José Gutiérrez Abascal 2, 28006 Madrid, Spain; Estación Biológica de Doñana (EBD), CSIC, Américo Vespucio 26, 41092 Seville, Spain; Estación Biológica de Doñana (EBD), CSIC, Américo Vespucio 26, 41092 Seville, Spain

**Keywords:** begging signals, communication, *Ficedula hypoleuca*, parent–offspring conflict, scramble competition, vitamin E

## Abstract

Parent–offspring conflict over food allocation can be modeled using two theoretical frameworks: passive (scramble competition) and active choice (signaling) resolution models. However, differentiating between these models empirically can be challenging. One possibility involves investigating details of decision-making by feeding parents. Different nestling traits, related to competitive prowess or signaling cryptic condition, may interact additively or non-additively as predictors of parental feeding responses. To explore this, we experimentally created even-sized, small broods of pied flycatchers and manipulated nestling cryptic quality, independently of size, by vitamin E supplementation. We explored how interactions between nestling cryptic condition, size, signals, and spatial location predicted food allocation and prey-testing by parents. Parents created the potential for spatial scramble competition between nestlings by feeding from and to a narrow range of nest locations. Heavier supplemented nestlings grew faster and were more likely to access profitable nest locations. However, the most profitable locations were not more contested, and nestling turnover did not vary in relation to spatial predictability or food supply. Postural begging was only predicted by nestling hunger and body mass, but parents did not favor heavier nestlings. This suggests that size-mediated and spatial competition in experimental broods was mild. Pied flycatcher fathers allocated food in response to nestling position and begging order, while mothers seemingly followed an active choice mechanism involving assessment of more complex traits, including postural intensity interacting with order, position, and treatment, and perhaps other stimuli when performing prey-testings. Differences in time constraints may underlie sex differences in food allocation rules.

## Teaser

Pied flycatcher moms allocated food to hungry chicks by integrating multiple nestling traits; dads responded swifter and less complexly. Parent birds may distribute food passively (to the most distinct competitor) or actively (by choosing among offspring signals). We manipulated nestling quality, independently of size, by supplementing vitamin E, and studied how parents responded to interacting nestling traits. Males favored closer, first-begging chicks while females assessed more complex traits (begging, prey-testing) to choose hungry nestlings.

## INTRODUCTION

Begging signals, including postures, vocalizations, and colorful integuments, play a significant role in parent-offspring feeding dynamics (Kilner and Johnstone 1997; Budden and Wright 2000; Wright and Leonard 2002). Theoretical work suggests that begging signals of nestling condition have evolved as a costly mechanism of resolution of parent–offspring conflict over the amount and distribution of food resources (Godfray 1991, 1995; Parker et al. 2002a, 2002b), but the precise behavioral mechanism underlying parental feeding remains debated. Early models suggest that begging is a form of scramble competition among siblings, with parents passively providing food to the offspring with the strongest signal through a simple, fixed mechanism (Macnair and Parker 1979; Godfray and Parker 1991). In contrast, recent models propose that begging is an honest signal, and parents actively choose to feed needier chicks after a careful scrutiny of offspring signals (Godfray 1991, 1995; Godfray and Johnstone 2000). Distinguishing empirically between passive and active feeding mechanisms is challenging because both models make similar predictions regarding how offspring condition affects begging performance and parental response (Parker et al. 2002a, 2002b; Royle et al. 2002, 2004).

Both models define control over food distribution as opposing ends of a continuum, but natural systems may exist anywhere along this spectrum (Royle et al. 2002). Studies on birds (Hinde et al. 2010; Lucass et al. 2016a) and insects (Smiseth et al. 2003a; Mäenpää and Smiseth 2020) have explored whether the rate and timing of total food transfer is under parent or offspring control. However, it is unclear how these findings apply to food distribution in multiple families, as provisioning and allocation may depend on different behavioral mechanisms (Johnstone 2004). Food allocation in bird families is a multifaceted process influenced by various nestling and parental traits (Krebs 2001). These include nestling size (Smiseth et al. 2003b), spatial location (Budden and Wright 2001), conspicuousness (Wiebe and Slagsvold 2009, 2012), and parental sex (Kilner 2002a, 2002b; Lessells 2002). Nestling position in the nest depends on offspring dynamics and it is widely considered as scramble competition (McRae et al. 1993; Budden and Wright 2001). Also, heavier nestlings are superior competitors: they can monopolize more profitable locations (Ostreiher 2001; Dickens et al. 2008) and provide stronger stimuli to parents (MacGregor and Cockburn 2002; Smiseth et al. 2003a; Wiebe and Slagsvold 2012). Several studies aimed to differentiate between passive (scramble) and active (signaling) models by investigating whether food distribution depends on nestling competitive traits or postural begging signals (Krebs 2001; Kilner 2002a; Smiseth and Amundsen 2002; Porkert and Špinka 2006; Tanner et al. 2007, 2008; Li et al. 2019). However, signals can serve as a form of competition (Macnair and Parker 1979; Godfray and Johnstone 2000; Kilner 2002a), while nestling competition may communicate information and aid parents in making active decisions (Lotem et al. 1999; Rodríguez-Gironés et al. 2001). Multiple broods should be viewed as offspring scrambles (between signals and positions), where parents either passively dole out to the most showy competitor or choose actively among different scramblers to spot the rightful chick. Some studies interpreted a parental preference for larger offspring, after controlling for nestling behavior, as evidence of active choice (Smiseth et al. 2003b; Shiao et al. 2009; Soler et al. 2022). However, these results are also consistent with food being passively delivered by parents in response to the greater overall begging stimulus posed by larger chicks, (Royle et al. 2004) (for a complementary experimental approach controlling parental behavior in insects, see Andrews and Smiseth 2013). Therefore, it is not always clear which nestling traits should we expect to be favored under a passive or active parental feeding scenario (Smiseth et al. 2003a, 2003b; Kölliker 2011).

Parker et al. (2002a, 2002b) proposed an alternative approach to differentiate between passive and active parental allocation based on parental decision-making processes related to begging, which require details of exactly how parents allocate food:

1) Passive feeding involves a fixed response to the nearest most-intense begging stimulus, which can include the amplifying effects of nestling position, size, and conspicuousness (Parker et al. 2002b). Quoting literally: […*the parent responds to the nearest most-intense stimulus, which one can imagine as a series of unequal, overlapping hemispheres, one for each chick, each with its intensity falling away from the centre of its base (the gape of the begging chick) towards its periphery*; p. 305]. Therefore, we predict nestling traits to have an additive or reinforcing interaction effect on parental preference (e.g., large nestlings that stretch more are most favored). Active choice, on the other hand, is expected to be more flexible, with the parent compensating for any amplification of begging signal, and allocating food based on offspring condition, particularly need/hunger (Parker et al. 2002a). Hence, we predict a conditional or non-reinforcing interaction between traits (e.g., intense begging is favored in spite of a small size or poor location). Ever since Parker et al. (2002a, 2002b), limited progress has been made in understanding how adult birds integrate various components of begging displays and make decisions related to begging (MacGregor and Cockburn 2002; Wiebe and Slagsvold 2012), partly due to the challenges of disentangling interaction effects between multiple correlated nestling traits and simultaneous brood mate behavior (Whittingham et al. 2003).
2) Under parental active choice, […*one would expect some form of comparison of the hemispheres, then a return to the largest after compensation for the effect of size of the offspring* (Parker et al. 2002b, p. 305]. One indication of active assessment of begging signals is if parents check each offspring’s begging level in turn before returning to feed the hungriest offspring (Royle et al. 2002). Passerine birds usually provide only one food item per visit, blurring the distinction between active and passive feeding (Kilner 2002a). However, some parents perform prey-testing by placing prey on the gapes of multiple nestlings and withdrawing it before allocating food to one (Forbes 2007; Wiebe and Slagsvold 2012; Stalwick and Wiebe 2019). Prey-testing is a poorly understood behavior that may assist parents in feeding decisions (Wilson and Clark 2002; Morales and Velando 2018), and it could indicate active parental choice if food allocation is based on need or hunger instead of competitive ability. Active choice models predict parental preference for needier offspring, particularly if they are equal or poorer competitors (Royle et al. 2002, 2004; Fresneau and Müller 2016), such as when larger front nestlings are bypassed in favor of hungrier siblings (Smith et al. 2017).

In this study, our aim is to test the predictions proposed by Parker et al. (2002a, 2002b) for the first time. We postulate that examining additive and non-additive fixed effects in multiple linear regression models can provide statistical evidence of how interactions among nestling traits affect feeding responses of pied flycatcher parents (*Ficedula hypoleuca*). Pied flycatchers exhibit intricate feeding patterns, integrating various nestling traits in ways that vary with parental sex (Gottlander 1987; Khayutin et al. 1988; Wiebe and Slagsvold 2012). To circumvent the confounding effects of nestling size on parental preferences (see above), we induced variation in nestling cryptic quality, independently of size, by supplementing nestlings with vitamin E. Vitamin E has positive effects on nestling growth, which suggests preferential food allocation toward the supplemented nestlings (De Ayala et al. 2006; Marri and Richner 2014; Montoya et al. 2020). Vitamin E also reduces telomere attrition (Pérez-Rodríguez et al. 2019; Pineda-Pampliega et al. 2020) and enhances immune response (Niu et al. 2009), and probability of fledging (Maronde and Richner 2015). Thus, supplementation may highlight variation in nestling cryptic condition, potentially conveyed through signals such as begging (Noguera et al. 2010) or coloration (Bertrand et al. 2006; Leclaire et al. 2011; Martínez-Renau et al. 2021). Second, we removed any bias in nestling size caused by experimental treatment, and prevented despotic interactions between chicks, by creating small (4-chick) broods of even-sized nestlings that were video recorded under natural conditions.

We made the following predictions for our study: 1) parents will favor supplemented nestlings due to their more efficient signals or competitive ability. 2) When parents are passively allocating food, nestling traits related to a higher competitive ability (position, relative size, conspicuousness, or begging performance) will have an additive or synergic effect on parental preference. 3) When parents are actively choosing which nestling to feed, we expect non-additive (negative or conditional) interactions between nestling traits, particularly if 3) parents favor needier or supplemented offspring with equal or less competitive ability. If prey-testing is a mechanism of active choice by which parents acquire information about nestling hunger, they will 4) use it mainly when they are uncertain about variations in hunger (i.e., when more chicks beg simultaneously with similar intensities) and 5) eventually allocate food to hungrier chicks.

## METHODS

### Experimental setup

The study was conducted in 2016 within a population of pied flycatchers in an old oak forest (*Quercus pyrenaica*) located in La Hiruela (Madrid, Spain; 41°04N 3°27W). Five days after hatching, nestlings were ranked by their mass and assigned to either a vitamin E supplementation or control treatment, alternating the order of treatments between successive broods to ensure an unbiased sample with respect to hatching order and balanced across different brood sizes. Supplemented nestlings received a dipteran larva soaked in a Vitamin E solution dissolved in organic coconut oil, following a previously established protocol in the same study population (Pérez-Rodríguez et al. 2019). Control nestlings received a larva soaked in coconut oil. All nestlings received a new supplementation every other day, starting when 5 days old and were weighed to the nearest 0.01 g. Ethical approval was granted by “Consejería de Medio Ambiente y Ordenación del Territorio” (Comunidad de Madrid) (Ref. PROEX 117/15). Further details are provided in the Supplementary Methods.

### Video and image recordings

We recorded video sequences of parent and chick behavior when broods were 7 days old. On the morning of day 7, the four nestlings of each brood with the most similar body masses (two of each treatment) were fed to satiation with one to five larvae to equalize food deficit (Wright et al. 2002) and marked individually. We played recorded video sequences at 1/4 speed to accurately measure the behavior of parents and chicks during each parental feeding visit. A trained observer who was blind to the experiment’s aim recorded behavioral rates. During each parental visit, we distinguished between parental feeding and prey-testing behavior. We defined a feeding as a parent introducing a food item into the mouth of a nestling and the nestling swallowing it. By contrast, a testing involved a parent introducing its beak with prey into a nestling’s mouth but failing to release any food item (Slagsvold and Wiebe 2007).

We divided the nest cup into eight equal 45° circular sectors, with sector 1 being closest to the nest entrance, as previously described by Khayutin et al. (1988). During parental feeding visits, we recorded the location of each nestling, the sex and location of parents, the duration of the visit, the number of feedings and prey-testings received, the order in which nestlings begged (1 = first), and the maximum begging postural intensity scored on a five-level ordinal scale following Redondo and Castro (1992). To assess nestling mobility, we calculated the mean number of sector changes between parental visits. We also measured the time of food deprivation for each nestling during a given visit as the time elapsed since the previous visit when it was fed. To account for variation in initial deprivation times between nests, we computed the deprivation time for each nestling as a fraction of the maximum value of deprivation time for each brood during the entire recording session.

On day 7, we recorded the mouth coloration of 72 vitamin E and 81 control nestlings from 32 broods, following video sessions. From digital images collected under standard conditions, we quantified color descriptors (visible spectrum) of areas of interest. We measured the saturation value of nestling’s palate as an indicator of carotenoid pigmentation (Saino et al. 2003; Dugas and McGraw 2011), and lightness (total reflectance) of inner and outer parts of the mouth flanges as an indicator of conspicuousness (Kilner and Davies 1998; Wiebe and Slagsvold 2009) (See Supplementary Methods for further details).

### Statistical analyses

We analyzed the data using linear mixed effects models with restricted maximum likelihood, implemented with the “nlme” package (Pinheiro et al. 2020) in R 4.0.2 (R Development Core Team 2022). We used a top-down strategy to determine the optimal random- and fixed-effect structure for each model, by comparing nested models using likelihood ratio tests (Zuur et al. 2009). Please refer to Supplementary Table S1 for a description of all saturated models and see Supplementary Methods for further details.

To confirm that the experimental treatment had the intended effect on nestling mass gain, we compared the differences in nestling mass between treatments on days 7 and 9, while holding initial mass at day 5 as a covariate. To analyze the effect of the treatment on nestling flange conspicuousness, we conducted a principal component analysis (PCA) on the lightness values of the inner and outer areas of the flange using the function prcomp() from the “stats” package. We used the individual scores on the first principal component (PC1) as a measure of overall lightness, as they were highly correlated with the lightness values of both the inner and outer parts of the flange (PC1 loading value = 0.707). To examine the effect of vitamin E supplementation on nestling behavior, we constructed individual models for different variables including the probability of gaping, begging order, postural intensity, profitability of nestling spatial location, inter-feeding intervals, and rate of change between nest sectors. We included the sex of the parent as a fixed effect, as it was found to influence nestling behavior in some studies (Wetzel et al. 2020). We used a generalized linear mixed model with a binomial error distribution to analyze the probability that a nestling would gape in a given visit. Pied flycatcher parents favor nestlings located in specific nest positions (Gottlander 1987), resulting in those sectors being highly profitable for the chicks, while the remaining sectors had a low or zero probability of food acquisition. We computed spatial profitability as the fraction of total feedings a parent delivered to a nestling’s sector relative to the location of the feeding parent during a given visit. Additionally, as a measure of nestling spatial mobility (jockeying, McRae et al. 1993) we calculated the rate of change between nest sectors by dividing the number of sector changes made by each nestling by the number of feeding visits. To investigate the relationship between nestling mobility and hunger levels, we calculated the mean time of food deprivation for each chick and the average rate of change between sectors per brood by dividing the mean number of position changes by nestlings for the entire brood by the number of feeding visits (McRae et al. 1993). Predictable, parentally favored sectors in the nest cup are considered the major driver of spatial scramble competition among nestlings (Kacelnik et al. 1995). Accordingly, we included the number of caring parents and the degree of overlap between parent sexes in the use of nest sectors as fixed effects in the model to test whether the predictability of parental feeding position influenced nestling mobility (Kölliker and Richner 2004). We calculated the degree of overlap between parent sexes in the use of nest sectors for a given brood as the difference between the total number of feedings given from sectors shared by both parents minus the number of feedings given from non-overlapping sectors, divided by the total number of feeding events.

We used generalized linear mixed models with a binomial error distribution to analyze parental feeding preferences. Since allocation patterns may differ according to parental sex (Kilner 2002b; Ryser et al. 2016), analyses were conducted separately for females and males. To prevent issues with model convergence due to a high number of parameters being estimated simultaneously, the analysis was conducted in two steps; the first used all nestling traits as predictors, while the second built models using only significant predictors and included pairwise interactions between them, as well as with relative body mass (Supplementary Table S1). Since differences between females and males in their degree of familiarity with nestlings could explain differences in allocation rules (Gottlander 1987; Lucass et al. 2016b), we also analyzed sex differences in the feeding rate of parents and the duration of each feeding visit (Supplementary Table S1).

We proceeded in three steps to explore prey-testing behavior by parents. Firstly, we searched for differences between visits where testing occurred and those where it did not. Secondly, in a given visit, we compared the behavior of nestlings that were either tested, fed or neither fed nor tested. Finally, we examined whether the probability of being fed after having been tested varied according to nestling body mass and condition (treatment and time of food deprivation). Supplementary Methods provide further details.

## RESULTS

Body mass of nestlings on days 7 and 9 was significantly affected by the interaction between experimental treatment and initial body mass. Nestlings supplemented with vitamin E showed a stronger positive relationship between mass on day 5 and mass on days 7 or 9 compared to control nestlings (β ± SE = 0.262 ± 0.069, *t*_1, 100_ = 3.791, *P* < 0.001; β ± SE = 0.339 ± 0.101, *t*_1, 89_ = 3.358, *P* = 0.001, respectively) (Supplementary Figure S1 and Supplementary Table S2). For the subsample of nestlings selected for video recordings at day 7, there was no significant difference in body mass between supplemented and control nestlings (β ± SE = −0.374 ± 0.292, *t*_ 1, 38_ = −1.279, *P* = 0.209), thus excluding any potential mass bias between treatments for this subsample. Our brood size manipulation however did not remove variation in nestling size, both within and between broods. The mean intra-brood mass range (heavier-lighter), expressed as a fraction of average body mass, was 0.28 g (± 0.03 SE), ranging from 0.10 g (9.91–10.93 g) to 0.70 g (5.18–11.72 g).

### Nestling signaling and spatial dynamics

Hungrier and heavier nestlings tended to beg earlier and more often when a parent entered the nest box. Nestling gaping probability and begging order were predicted by deprivation time and relative body mass, but not by the experimental treatment (Table 1). Additionally, begging postural intensity was affected by time of food deprivation and relative body mass, as well as an interaction between hatching date and treatment (Table 1). Control nestlings showed a decrease in begging postural intensity with increasing hatching date, while postural intensity of nestlings supplemented with vitamin E did not exhibit a similar seasonal trend. Nonetheless, the postural intensities of nestlings from both treatments were similar overall (Supplementary Figure S2). Mouth coloration (palate color saturation and flange lightness) was not significantly affected by the experimental treatment (Supplementary Table S3). However, palate saturation increased with hatching date (β ± SE = 0.012 ± 0.005, *t*_ 1, 30_ = 2.624, *P* = 0.014).

**Table 1:** Estimated parameters (± SE), *z* values for models explaining the probability of a nestling to beg and *t* values for models explaining nestling begging order, postural intensity, spatial profitability, and rate of change between nest sectors

	Estimate	SE	*z*/*t*	*P*
Probability to beg^a^				
Intercept	0.522	0.173	3.011	**0.003**
Deprivation time	0.304	0.060	5.030	**<0.001**
Body mass	0.182	0.080	2.260	**0.024**
Treatment	0.071	0.157	0.452	0.652
Begging order^b^				
Intercept	3.174	0.096	32.96	**<0.001**
Deprivation time	−0.230	0.035	−6.531	**<0.001**
Body mass	−0.215	0.097	−2.206	**0.044**
Treatment	−0.029	0.119	−0.242	0.809
Postural intensity^c^				
Intercept	1.066	0.105	10.14	**<0.001**
Treatment × Hatching date	0.164	0.069	2.385	**0.020**
Hatching date	−0.155	0.092	−1.683	0.103
Deprivation time	0.125	0.026	4.831	**<0.001**
Parent sex	−0.114	0.062	−1.834	0.067
Body mass	0.105	0.049	2.163	**0.045**
Treatment	0.054	0.070	0.776	0.441
Spatial Profitability^d^				
Intercept	−2.148	0.129	−16.69	**<0.001**
Treatment × Body mass	0.198	0.096	2.069	**0.042**
Body mass	−0.081	0.074	−1.092	0.279
Deprivation time	−0.052	0.020	−2.569	**0.010**
Treatment	0.044	0.074	0.592	0.556
Rate of change between nest sectors^e^				
Intercept	0.386	0.029	13.24	**<0.001**
Brood size	0.064	0.027	2.356	**0.027**
Average deprivation time	−0.021	0.014	−1.524	0.131
Treatment	−0.011	0.023	−0.502	0.617

“Treatment” and “Parent sex” are categorical variables with “*Control*” and “*Male*” as the reference groups. Significant *P*-values are indicated in bold.

^a^Predictors (parent sex, brood size, and hatching date) and their interactions (Supplementary Table S1) that failed to improve model fit according to a likelihood ratio test are not depicted.

^b^Predictors (parent sex, brood size, and hatching date) and their interactions (Supplementary Table S1) that failed to improve model fit according to a likelihood ratio test are not depicted.

^c^Predictors (brood size) and their interactions (Supplementary Table S1) that failed to improve model fit according to a likelihood ratio test are not depicted.

^d^Predictors (parent sex, brood size, and hatching date) and their interactions (Supplementary Table S1) that failed to improve model fit according to a likelihood ratio test are not depicted.

^e^Predictors (parental feeding rate, body mass, and hatching date) and their interactions (Supplementary Table S1) that failed to improve model fit according to a likelihood ratio test are not depicted.

The experimental treatment and relative body mass interacted to affect the spatial profitability of nestling positions in the nest cup (Table 1). Heavier supplemented nestlings were more likely to occupy highly profitable positions than heavier control nestlings (Supplementary Figure S3). Spatial profitability decreased with time of food deprivation, and hungrier nestlings tended to be positioned in less profitable positions when a parent entered the nest box (Table 1). Brood size significantly affected the average rate of change between different nest sectors, but the average time of food deprivation, the experimental treatment and parental feeding rates did not (Table 1). Nestlings coming from larger broods changed position more between successive parental visits. Nestling behavior was not affected by the sex of the parent feeding in each visit (Table 1).

### Parental feeding behavior

Out of the 28 recorded nests, both male and female parents fed in 18 nests, while only one parent fed in 10 nests (eight of which were females and two were males). Female parents fed at a higher rate (8.01 visits/h ± 0.55 SE) than males (5.90 visits/h ± 0.91 SE), especially later in the season (interaction of parental sex with hatching date, β ± SE = 2.808 ± 0.944, *t*_ 1, 42_ = 2.973; *P* = 0.004). The interval between two successive parental visits was 3.94 min (± 0.39 SE) and 4.44 min (± 0.46 SE) for bi- and uniparental nests, respectively. During feeding visits, female parents stayed in the nest box for twice as long as males did (mean feeding visit duration ± SE = 29.32 ± 2.407 s vs. 12.49 ± 0.806 s, respectively; β ± SE = 0.604 ± 0.086, *t*_ 1, 584_ = 7.061; *P* < 0.001). The time interval between two consecutive feedings did not differ between supplemented and control nestlings, hence both groups experienced similar durations of food deprivation. Body mass was the only significant predictor explaining inter-feeding intervals. Heavier nestlings were fed more often than their lighter nest mates (β ± SE = −1.723 ± 0.549, *t*_ 1, 69_ = −141, *P* = 0.002) (Supplementary Table S4).

**Figure 1:**
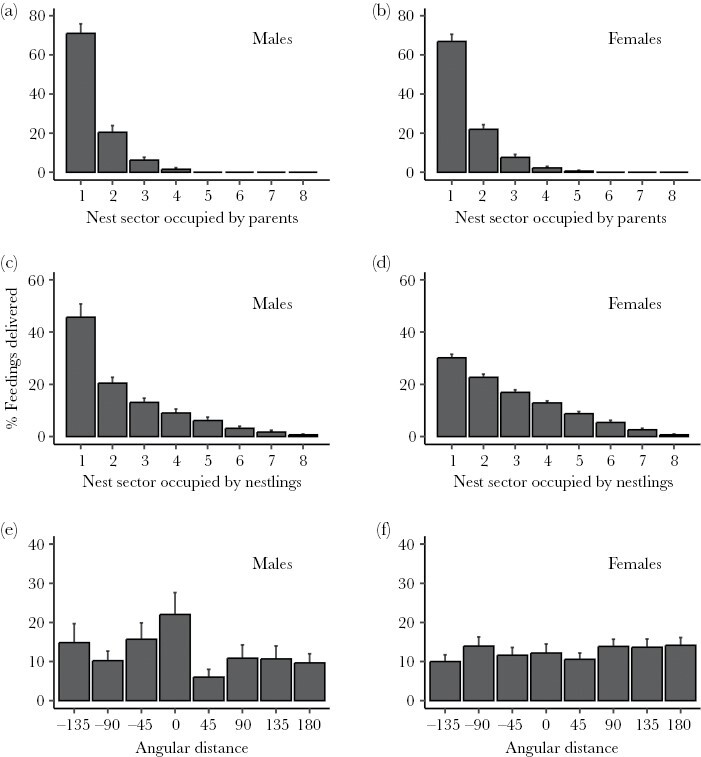
The distribution of feedings according to the position (nest sectors) occupied by parents (A, B), occupied by nestlings relative to parental position (C, D) and the angular deviation between parent and offspring position (E, F) ordered by frequency (A-D) or angular distance (E, F). Shown are percentages of feedings (mean ± SE) by males (A, C, E) and females (B, D, F).

We investigated how parental allocation patterns according to chick position could affect nestling spatial dynamics related to spatial scramble competition. Parents exhibited a clear tendency to feed chicks from a few favorite positions in the nest box. On average, males and females fed chicks from two preferred nest sectors from which more than 80% of all feedings were given (Figure 1A,B). While both parents used identical sectors in 3 out of 18 nests (16.7 %), in 11 nests (61.1 %) they shared at least 50% of their feeding positions. Moreover, parents exhibited strong preferences for feeding chicks positioned in a few favored locations relative to their own position. Typically, more than 60% of all feedings accumulated in only two (males) or three (females) different locations (Figure 1C,D). This preference was not simply a function of the angular distance between parents and chicks (Figure 1E,F). Males tended to feed chicks positioned very close to them (angular distance = 0), while females allocated feedings evenly irrespective of angular distance. In 43.8% of the visits (± 23.9 SE, *N* = 28 broods), at least one of the two most profitable nest sectors of the nest was vacant when the parent entered the nest box. Mean (brood) rates of change between nest sectors by nestlings were not significantly explained by either brood size, number of feeding parents, or the degree of overlap between parental feeding locations (Supplementary Table S5).

**Figure 2:**
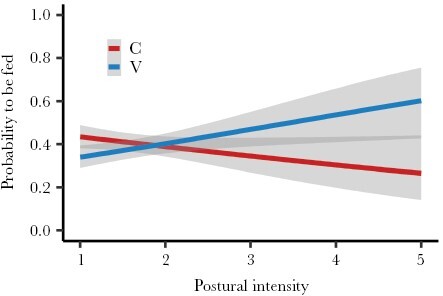
The effect of experimental treatment (V = vitamin E; C = control) and nestling postural intensity on the probability of being fed by female parents. Shown are observed regression lines and 95% CI (gray bands).

**Figure 3:**
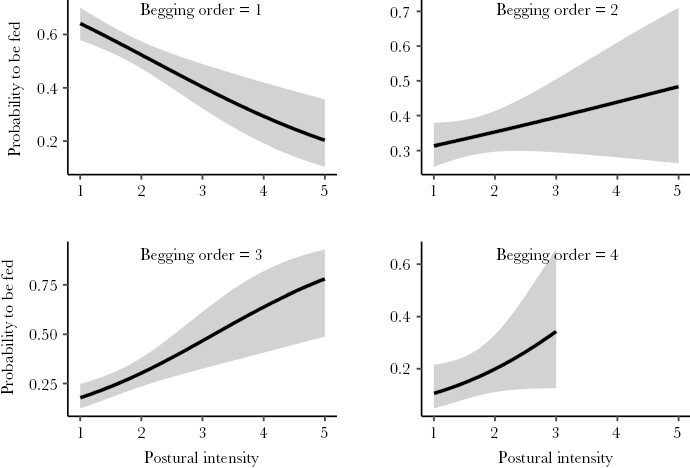
The effect of nestling begging order and postural intensity on the probability of being fed by female parents. Shown are observed regression lines and 95% CI (gray bands).

**Figure 4:**
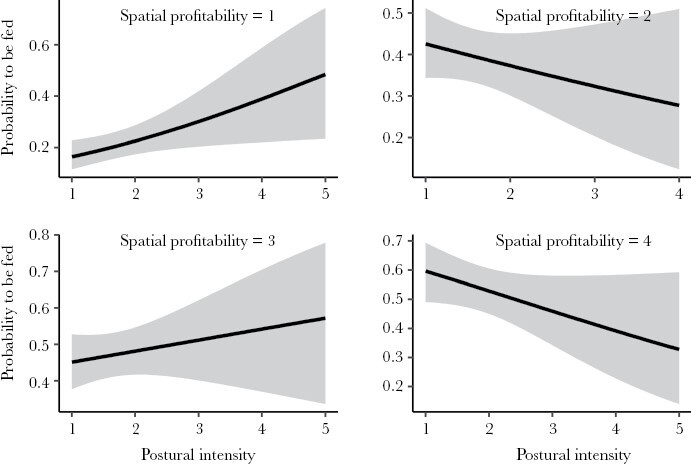
The effect of nestling spatial profitability and postural intensity on the probability of being fed by female parents. Shown are observed regression lines and 95% CI (gray bands).

Males and females exhibited distinct feeding preferences in relation to nestling characteristics. Male preferences were driven by nestling position and begging order, while female preferences were influenced by nestling position, begging order, and postural intensity interacting with experimental treatment (Table 2). In a second step, a model including all significant nestling traits and relative body mass revealed that male feeding was primarily influenced by nestling position and begging order, with a marginally significant effect of relative body mass. There were no significant interactions between predictors (Table 3). In contrast, female feeding preferences depended on a triple interaction of postural intensity with begging order, experimental treatment, and spatial profitability (Table 3). Accordingly, females showed a preference for supplemented nestlings with high postural intensities, but no preference for treatment was observed with low postural intensities (Figure 2). Nestlings positioned in more profitable sectors and those that started begging sooner were more likely to be fed by females at lower postural intensities, but females also favored nestlings in stretched postures begging later or in less profitable positions (Figures 3 and 4).

**Table 2:** Estimated parameters (± SE), and *z* values for models explaining feeding preferences (probability of feeding a particular nestling in a visit) of male and female parents according to nestling size, behavior, coloration, and vitamin E supplementation

	Estimate	SE	*z*	*P*
Males^a^				
Intercept	−0.353	0.139	−2.540	**0.011**
Spatial profitability	0.834	0.103	8.108	**<0.001**
Begging order	−0.702	0.111	−6.303	**<0.001**
Body mass	0.179	0.102	1.765	0.078
Treatment	−0.142	0.192	−0.739	0.460
Females^b^				
Intercept	−0.511	0.105	−4.872	**<0.001**
Spatial profitability	0.717	0.122	5.878	**<0.001**
Begging order	−0.711	0.099	−7.213	**<0.001**
Treatment × Postural intensity	0.452	0.144	3.135	**0.002**
Postural intensity	−0.255	0.108	−2.369	**0.018**
Treatment × Spatial profitability	−0.235	0.152	−1.549	0.121
Body mass	0.163	0.080	2.028	**0.043**
Palate saturation	−0.113	0.075	−1.502	0.133
Treatment	−0.084	0.148	−0.563	0.574

“Treatment” is a categorical variable with “*Control*” as the reference group. Significant *P*-values are indicated in bold.

^a^Predictors (postural intensity, palate saturation, flanges lightness PC1, brood size, and hatching date) and their interactions (Supplementary Table S1) that failed to improve model fit according to a likelihood ratio test are not depicted.

^b^Predictors (flanges lightness PC1, brood size, and hatching date) and their interactions (Supplementary Table S1) that failed to improve model fit according to a likelihood ratio test are not depicted.

**Table 3:** Estimated parameters (± SE), and *z* values for models explaining feeding preferences (probability of feeding a particular nestling in a visit) of male and female parents considering only significant predictors (begging behavior, position, relative body mass, and treatment) and all pairwise interactions between them

	Estimate	SE	*z*	*P*
Males^a^				
Intercept	−0.372	0.142	−2.618	**0.009**
Spatial profitability	0.883	0.124	7.133	**<0.001**
Begging order	−0.685	0.119	−5.756	**<0.001**
Body mass	0.191	0.102	1.862	0.063
Postural intensity	0.147	0.098	1.503	0.133
Treatment	−0.139	0.198	−0.703	0.482
Females^b^				
Intercept	−0.403	0.106	−3.787	**<0.001**
Begging order	−0.702	0.085	−8.263	**<0.001**
Spatial profitability	0.592	0.089	6.656	**<0.001**
Begging order × Postural intensity	0.428	0.084	5.080	**<0.001**
Treatment × Postural intensity	0.399	0.146	2.742	**0.006**
Treatment × Body mass	0.247	0.170	1.455	0.146
Spatial profitability × Postural intensity	−0.188	0.072	−2.601	**0.009**
Treatment	−0.179	0.150	−1.194	0.232
Postural intensity	−0.127	0.107	−1.185	0.236
Brood size	−0.107	0.073	−1.462	0.144
Body mass	−0.045	0.139	−0.325	0.745

“Treatment” is a categorical variable with “*Control*” as the reference group. Significant *P*-values are indicated in bold.

^a^Predictors (palate saturation, flanges lightness PC1, brood size, and hatching date) and their interactions (Supplementary Table S1) that failed to improve model fit according to a likelihood ratio test are not depicted.

^b^Predictors (palate saturation, flanges lightness PC1, and hatching date) and their interactions (Supplementary Table S1) that failed to improve model fit according to a likelihood ratio test are not depicted.

### Prey-testing by parents

We investigated whether prey-testing events by parents could indicate active choice (see Introduction). In total, we recorded 173 prey-testing events in 111 out of 656 feeding visits (16.9%). Prey-testing was widespread. Testing occurred in 25 out of 28 broods (89.3%), involving most of the parents observed: 75.0% (15/20 individuals) of males tested at least one chick in 34 feeding events, while 92.3% (24/26 individuals) of females tested chicks in 77 events. Females tested chicks significantly more often than males (χ^2^ = 40.787, *P* < 0.001). Prey-testing usually involved one (77.5% of testing events) or two different nestlings (16.2%), rarely three (5.4%) or four (0.9%).

Prey-testing events were more likely to occur during feeding visits where a higher fraction of nestlings begged at the same time (Supplementary Table S6). Nestlings tested by either parent were positioned in less profitable nest locations but had similar postural intensities as those fed (Table 4). Chicks tested by females were less hungry than fed nestlings, but no significant differences in deprivation time were detected between chicks tested and fed by males (Table 5). Additionally, an interaction between testing and experimental treatment was observed for nestling mass, where supplemented chicks that were tested tended to be smaller than those not tested, but no differences were detected among control chicks (Table 4, Supplementary Figure S4). Finally, the probability of being fed after being tested by a female parent was influenced by nestling hunger and relative mass, with tested chicks being more likely to be fed if they had been food deprived for longer (β ± SE = 0.711 ± 0.325, *z* = 2.191, *P* = 0.029) or had a higher body mass (β ± SE = 0.905 ± 0.394, *z* = 2.297, *P* = 0.022). There was no significant effect of the experimental treatment on this probability (β ± SE = 0.626 ± 0.643, *z* = 0.975, *P* = 0.330). A similar model for male testing events failed to converge.

**Table 4:** Estimated parameters (± SE), and *t* values for models explaining differences in begging order, postural intensity, spatial profitability, and relative body mass between nestlings according to parental testing

	Estimate	SE	*t*	*P*
Begging order^a^				
Intercept	1.861	0.106	17.59	**<0.001**
Testing				
Fed	−0.161	0.102	−1.578	0.115
Neither fed nor tested	0.401	0.100	4.000	**<0.001**
Neither fed nor tested × Hatching date	−0.262	0.107	−2.461	**0.014**
Fed × Hatching date	−0.252	0.108	−2.330	**0.020**
Hatching date	0.235	0.105	2.243	**0.025**
Treatment	−0.085	0.077	−1.099	0.275
Deprivation time	−0.059	0.025	−2.384	**0.017**
Postural intensity^b^				
Intercept	1.547	0.119	13.05	**<0.001**
Testing				
Fed	0.137	0.082	1.686	0.092
Neither fed nor tested	0.013	0.082	0.157	0.875
Parent sex	−0.120	0.061	−1.971	0.050
Treatment	0.089	0.067	1.340	0.185
Spatial profitability^c^				
Intercept	0.142	0.010	14.41	**<0.001**
Testing				
Fed	0.036	0.011	3.242	**0.002**
Neither fed nor tested	−0.003	0.009	−0.371	0.713
Fed × Deprivation time	0.005	0.008	0.543	0.588
Neither fed nor tested × Deprivation time	−0.009	0.008	−1.040	0.300
Body mass	0.007	0.004	1.768	0.081
Deprivation time	−0.004	0.008	−0.560	0.576
Treatment	0.003	0.008	0.356	0.723
Body mass^d^				
Intercept	0.134	0.161	0.831	0.406
Treatment	−1.675	0.237	−7.055	**<0.001**
Fed × Treatment	1.596	0.258	6.189	**<0.001**
Neither fed nor tested × Treatment	1.329	0.253	5.250	**<0.001**
Deprivation time	−0.257	0.127	−2.016	**0.044**
Fed × Deprivation time	0.114	0.137	0.834	0.405
Neither fed nor tested × Deprivation time	0.220	0.135	1.633	0.103
Testing				
Fed	0.108	0.176	0.611	0.541
Neither fed nor tested	0.086	0.173	0.497	0.620

“Testing” is a three-level factor with three levels (“*Tested*,” “*Fed,*” and “*Neither fed nor tested*” chicks). “Treatment,” “Testing,” and “Parent sex” are categorical variables with “*Control*,” “*Tested nestlings,*” and “*Male*” as the reference groups, respectively. Significant *P*-values are indicated in bold.

^a^Predictors (parent sex, body mass, and brood size) and their interactions (Supplementary Table S1) that failed to improve model fit according to a likelihood ratio test are not depicted.

^b^Predictors (deprivation time, body mass, brood size, and hatching date) and their interactions (Supplementary Table S1) that failed to improve model fit according to a likelihood ratio test are not depicted.

^c^Predictors (parent sex, brood size, and hatching date) and their interactions (Supplementary Table S1) that failed to improve model fit according to a likelihood ratio test are not depicted.

^d^Predictors (parent sex, brood size, and hatching date) and their interactions (Supplementary Table S1) that failed to improve model fit according to a likelihood ratio test are not depicted.

**Table 5:** Estimated parameters (± SE), and *t* values for models explaining differences in food deprivation time between nestlings according to parental testing

	Estimate	SE	*t*	*P*
Males^a^				
Intercept	1.187	0.199	5.977	**<0.001**
Testing				
Fed	0.160	0.184	0.870	0.385
Neither fed nor tested	0.096	0.183	0.526	0.599
Treatment	−0.063	0.108	−0.583	0.564
Females^b^				
Intercept	1.203	0.132	9.104	**<0.001**
Testing				
Fed	0.248	0.126	1.964	**0.049**
Neither fed nor tested	0.153	0.127	1.211	0.226
Fed × Body mass	−0.194	0.104	−1.871	0.062
Neither fed nor tested × Body mass	−0.006	0.094	−0.069	0.945
Body mass	−0.055	0.094	−0.587	0.558
Treatment	0.016	0.095	0.164	0.871

“Testing” is a three-level factor with three levels (“*Tested*,” “*Fed,*” and “*Neither fed nor tested*” chicks). “Treatment” and “Testing” are categorical variables with “*Control*” and “*Tested nestlings*” as the reference groups, respectively. Significant *P*-values are indicated in bold.

^a^Predictors (parent sex, deprivation time, body mass, brood size, and hatching date) and their interactions (Supplementary Table S1) that failed to improve model fit according to a likelihood ratio test are not depicted.

^b^Predictors (parent sex, deprivation time, brood size, and hatching date) and their interactions (Supplementary Table S1) that failed to improve model fit according to a likelihood ratio test are not depicted.

## DISCUSSION

### Signaling and scramble competition in experimental broods

Nestlings given vitamin E had a stronger positive relationship (β = 0.3 g) between their mass on day 5 and mass on days 7–9 compared to control nestlings, suggesting a higher marginal mass gain, particularly for initially heavier nestlings. A previous study using the same protocol found that supplemented chicks grew faster, attained higher asymptotic mass, and showed reduced telomere attrition (Pérez-Rodríguez et al. 2019). Since these traits are predictive of subsequent survival in this and other species (Potti et al. 2002; Moreno et al. 2005; Boonekamp et al. 2014), supplemented nestlings were of a higher quality (Mock et al. 2011). Faster growth could be the result of alleviated physiological constraints from antioxidants (Marri and Richner 2014) or increased parental food allocation elicited via begging (Noguera et al. 2010; Maronde et al. 2018), coloration (Leclaire et al. 2011) or spatial competition.

Vitamin E supplementation did not affect visible mouth coloration, but it may have influenced UV reflectance, which was not quantified by our system, as shown in spotless starlings (*Sturnus unicolor*) (Martínez-Renau et al. 2021). Supplementation did not affect motor begging performance (begging order, probability of gaping, and postural intensity), in agreement with most previous studies (Hall et al. 2010; Noguera et al. 2010; Maronde and Richner 2015; but see Maronde et al. 2018). However, it may have increased vocal components of the begging display, which were not measured in this study (Noguera et al. 2010). Only nestling size and time of food deprivation explained begging performance. Hungrier and heavier nestlings were more likely to gape, begged sooner and in more stretched postures. The spatial behavior of nestlings was predicted by an interaction between body mass and experimental treatment. When a parent arrived with food, it was more likely to find a large, supplemented chick in one of its favorite locations, rather than a small supplemented one or a control chick. Jockeying for favorable positions is mediated by learning and maturation of sensorimotor abilities (Budden and Wright 2005), and older (bigger) nestlings may compete more efficiently (Kilner 1995; Slagsvold and Rohwer 2000). Although supplemented nestlings in experimental broods were not consistently heavier than controls, they were growing faster and may have matured earlier.

During feeding, parents exhibited a strong preference for certain nest sectors and feeding locations, creating the potential for spatial scramble competition between chicks (Gottlander 1987; Kilner 1995; Dickens et al. 2008). However, evidence suggests that spatial scramble competition in the studied broods was mild during the observation period. First, one of the two most profitable locations was vacant in 44% of visits, indicating that profitable positions were not contested more frequently than other positions (Dearborn 1998; Porkert and Špinka 2006). Second, large control and small chicks occupied similar positions regardless of body mass, unlike what is expected under intense spatial competition (Ostreiher 1997, 2001). Third, parental feeding predictability (e.g., number of feeding parents or the degree of overlap in parental feeding locations) did not influence jockeying between visits, as predicted by previous studies (McRae et al. 1993; Kölliker and Richner 2004; Tanner et al. 2007). Lastly, nestling movement rates did not correlate with food supply proxies (average time of food deprivation or parental feeding rate) (McRae et al. 1993; Porkert and Špinka 2006). In several studies, hungrier nestlings move toward more profitable nest locations (Kilner 1995, Porkert and Špinka 2006, Dickens et al. 2008; but see Smith and Montgomerie 1991). However, the overall nestling turnover may actually be caused by the less hungry nestlings moving actively from more to less profitable positions (Porkert and Špinka 2006). Spatial profitability in this study decreased with time of food deprivation, so nestlings in more profitable positions were more satiated. This is likely a result of intervals between chick position changes being longer than intervals between parental visits (Porkert and Špinka 2006). In pied flycatchers, changes in chick position may also take longer (30 min for 8-day broods, Khayutin et al. 1988) than intervals between feeding visits (4 min, this study). While spatial turnover is often thought of as a purely competitive mechanism (Kölliker and Richner 2004), other explanations are possible, such as cooperative coordination among chicks (Wilson and Clark 2002; Royle et al. 2012).

Brood size manipulation successfully removed any size bias due to experimental treatment, but not intra-brood variation in nestling mass. Parents, however, did not show any preference for heavier nestlings. Both parents allocated food to nestlings begging sooner. Mothers also favored nestlings begging in more stretched postures, particularly when supplemented with vitamin E. Despite heavier nestlings begged sooner and more intensively, we failed to detect any reinforcing interactions between body mass and begging or spatial cues on parental preferences. Heavier nestlings were fed more often than their lighter nestmates, suggesting that they were actually treated as hungrier. Larger nestlings may need more food in the short-term, be less constrained to use food resources for growth, and retain food in the digestive system for a shorter time, and as a result, larger nestlings may become hungry faster than smaller ones (Dickens et al. 2008). Thus, size asymmetries in experimental broods contributed little to scramble competition for more conspicuous begging stimuli.

Summarizing, food distribution was primarily predicted by offspring traits under offspring control (begging performance and position), but we lack evidence that scramble competition was the primary determinant of food distribution. Rather than simply accepting the outcome of nestling interactions (Davis et al. 1999), parents may actively control allocation by modifying their feeding decisions and establishing independent competition rules for access to their respective favored sectors (Kölliker et al. 1998; Kölliker and Richner 2004). For example, females in this study were less predictable than males in allocating food according to nestling position, they used a wider range of sectors to feed, and their favored positions were not simply based on the distance to nestlings relative to their own position.

### When do parents show passive and active feeding?

Males fed based on nestling position and begging order; females on nestling position, begging order, and postural intensity interacting with experimental treatment. However, such differences do not indicate active or passive feeding by parents. For example, both parents could be responding passively to different combinations of nestling traits. The key distinction between passive and active choice feeding models is how parents integrate the information coming from different nestling traits and whether this process results in different signals being evaluated (Parker et al. 2002a, 2002b) (see Introduction). Male pied flycatchers responded strongly to nestling position (β = 0.88) and, secondly, to begging order (β = −0.68) in a simple, additive way, being apparently oblivious to postural intensity. This pattern is consistent with predictions of a passive decision model where fathers simply add up effects of quickly assessable cues. Females responded to a double, non-reinforcing interaction between postural intensity and begging order (β = 0.43), and, less importantly, nestling position (β = −0.19). This pattern is consistent with active choice where effects of latency and position are discounted from effects of postural intensity (Kilner 2002a; Tanner et al. 2008). Mothers also showed a relatively important (β = 0.40), reinforcing interaction between postural intensity and experimental treatment, favoring supplemented over control nestlings when both begged intensively. This suggests that mothers might assess cryptic nestling quality, possibly via UV visual signals (Martínez-Renau et al. 2021).

Our results support predictions of the hypothesis that prey-testing is a back-up mechanism by which parents (particularly females) can assess the actual hunger levels of nestlings independently of postural begging (Wilson and Clark 2002; Fresneau and Müller 2016). For example, they could use tactile cues if hungrier nestlings attempted to swallow food faster or with a greater gape pressure. First, prey-testing occurred in visits where a higher number of nestlings begged simultaneously with similar postural intensities. Second, tested nestlings begged with similar postural intensities but were less hungry than fed nestlings. Differences in postural intensity between nestlings can be more difficult to assess than differences in latency or position, especially when nestlings differ in size (Rodríguez-Gironés et al. 2002; Fresneau et al. 2018). Hence, adults may be uncertain about nestling hunger when many chicks beg simultaneously with similar intensities. Interestingly, in other studies, the category of nestlings that was tested more often was also the one that begged most (e.g., brood parasites, Turtumøygard and Slagsvold 2010, Soler et al. 2017; UV-blocked nestlings Morales and Velando 2018). Finally, a tested chick was more likely to be eventually fed by a female if it was hungrier than its nestmates. Females also tested chicks more often than males. Thus, females seemed capable of active choice based on complex traits to gather accurate information about offspring condition. This was likely time-consuming, as feeding visits by females lasted for twice as long as those of males.

Our results suggest that, under the conditions of the study, male and female pied flycatchers are closer to opposite endpoints in the passive/active feeding continuum. This makes sense if males are more time constrained since, by definition, passive feeding is a swifter mechanism of allocation. Male pied flycatchers trade-off provisioning young with signaling for polygynous and extra-pair matings (Sanz 2001; Canal et al. 2012; Mänd et al. 2013), and male reliance on quickly assessable cues (position, latency, size) is widespread in the literature (Kilner 2002a, 2002b; Ryser et al. 2016). Patterns of feeding and prey-testing by females suggest active scrutiny of postural begging signals (and perhaps other stimuli). Females, however, can switch to more quickly assessable cues (size and position) when time-constrained, for example after an experimental enlargement in brood size (Budden and Beissinger 2009) or hunger (Gottlander 1987; Krebs and Magrath 2000; Kilner 2002b; Tarwater et al. 2009), or when unaided by helpers at the nest (Li et al. 2019).

## Supplementary Material

arad043_suppl_Supplementary_Material

## Data Availability

Analyses reported in this article can be reproduced using the data provided by Parejo-Pulido et al. (2023).
