# Supplementary material for: Passive and active parental food allocation in a songbird
Source: Behav Ecol. 2023 May 29;34(5):729–40. doi: 10.1093/beheco/arad043 (PMC10516681; doi:10.1093/beheco/arad043)
Supplement: arad043_suppl_Supplementary_Material [file arad043_suppl_supplementary_material.pdf]

**Title:** Passive and active parental food allocation in a songbird

**Short title:** Food allocation in bird broods

Daniel Parejo-Pulido[1], Lorenzo Pérez-Rodríguez[1], Inmaculada Abril-Colón[2], Jaime Potti[3] and Tomás Redondo[3]

1. Instituto de Investigación en Recursos Cinegéticos (IREC), CSIC-UCLM-JCCM, Ronda de Toledo 12, 13005 Ciudad Real, Spain

2. Museo Nacional de Ciencias Naturales (MNCN), CSIC, Departamento de Ecología Evolutiva, José Gutiérrez Abascal 2, 28006 Madrid, Spain

3. Estación Biológica de Doñana (EBD), CSIC, Américo Vespucio 26, 41092 Seville, Spain

**Corresponding author:** Tomás Redondo. Estación Biológica de Doñana (EBD), CSIC, Seville, Spain. +34 647994225. redondo@ebd.csic.es

## SUPPLEMENTARY METHODS

### Experimental setup

We monitored the nest boxes regularly to determine the exact start laying (± 24 h) and hatching dates (± 12 h; day 1 was considered the hatching day). Initially, we included 32 broods (174 nestlings) of monogamous pairs (Lundberg and Alatalo 1992) containing four to six nestlings that were five days old. Within each brood, we ranked the nestlings by mass and assigned them to either a vitamin E supplementation or control treatment, alternating the order between successive broods to ensure an unbiased sample with respect to hatching order and balanced across different brood sizes. As in a previous study conducted in the same population (Pérez-Rodríguez et al. 2019), supplemented nestlings were fed a dipteran larva (*Calliphora* sp.) soaked in a solution of vitamin E (DL-alpha-tocopherol acetate; Sigma-Aldrich ref. T3376) dissolved in organic coconut oil. The concentration of this vitamin E solution was adjusted across nestling development to provide a constant dose of 1.2 mg of vitamin E per kg per visit to the supplemented nestlings. Control nestlings were fed a larva soaked in coconut oil. Regardless of the treatment, nestlings received a new supplementation every other day, and we recorded their mass at each visit using an electronic balance (± 0.01 g). There was no significant difference in initial (day 5) body mass between supplemented and control nestlings ($\beta \pm$ SE = -0.232 ± 0.155, $t_{1, 133}$ = -1.500, $P$ = 0.136).

### Video recordings

We recorded video sequences of parent and chick behavior when the broods reached 7 days old. This age was selected since it corresponds to the peak of daily mass gain (Siikamäki 1996), and nestlings' feeding success still relies mostly on their begging behavior, before they can intercept food from adults at the nest entrance (Khayutin et al. 1988). On day 6, we attached a dummy camera to the roof of the nest-box to habituate

the birds. In the morning of day 7, we fed the four nestlings with the most similar body masses (two from each treatment) to satiation with 1-5 larvae to equalize food deficit (Wright et al. 2002). We marked each nestling's head with a unique combination of blue adhesive kinesiology tape badges varying in shape (circle vs. triangle) and markings (closed vs. open dots) (Porkert and Špinka 2006). The badges were randomly assigned with respect to nestling treatment and relative mass. We used a Sony Go-Pro video camera to obtain video recordings for as long as the camera batteries allowed (about 2 h). We obtained video samples from 28 nests. Meanwhile, we kept the surplus chicks from each brood in heated containers, hand-fed them every 30 min, and returned them to their natal nest after recording.

We played recorded video sequences at 1/4 speed to accurately measure the behavior of parents and chicks during each parental feeding visit. A trained observer, blind to the aim of the experiment, computed behavioral rates. During each parental visit, we distinguished between parental feeding and prey-testing behavior. We defined a feeding as a parent introducing a food item into the mouth of a nestling and the nestling swallowing it. Prey-testing involved a parent introducing its beak with prey into a nestling mouth but failing to release any food item (Slagsvold and Wiebe 2007). Both parents feed nestlings with prey of a similar size at this age, and nestlings can efficiently handle most prey (Slagsvold and Wiebe 2007; Wiebe and Slagsvold 2009a). However, we discarded testing events (four visits) where prey items were of an unusually large size, which could be explained by gape size constraints rather than parental choice (Wiebe and Slagsvold 2009a). We also recorded nestling behavior in a time window ranging from 10 seconds before the adult entered the nest box to 10 seconds after the adult left it, to record nestling behavior while adults remained poking at the nest box from the outside during each parental visit.

We divided the nest cup into 45° circular sectors, numbered clockwise from 1 to 8, to record parent and nestling behavior during feedings (Khayutin et al. 1988). We noted the sector from which each parent fed nestlings and the sector occupied by each nestling when parents entered the nest box. During each feeding visit, we recorded its duration, the spatial location of parents, the number of feedings and prey-testings received, the order in which nestlings begged (1 = first) and the maximum begging postural intensity of each nestling. We used a five-level ordinal scale to score the degree of body stretching as an indicator of begging postural intensity, following Redondo and Castro (1992). Additionally, we computed the rate of individual nestling movement as the average number of sector changes between parental visits. To calculate time of food deprivation for each nestling during a given visit, we measured the time elapsed since the previous visit when it was fed. Since initial deprivation times varied between nests, we computed deprivation times for each nestling as a fraction of the maximum value of deprivation time for each brood in the entire recording session.

Nestling mouth coloration

On day 7, we photographed the mouth coloration of 72 vitamin E and 81 control nestlings from 32 broods. We gently opened each nestling's mouth in a standardized position and captured images using a Nikon D80 digital camera with a Sigma AF 50/2.8 objective and consistent illumination. To ensure accurate color representation, we included a ColorChecker Passport Photo 2 (X-Rite, Regensdorf, Switzerland) color reference in a standard position in each image. Images in RAW format were processed using SpotEgg software (Gómez and Liñán-Cembrano 2017) to normalize and linearize them. We then used Adobe Photoshop CS6 v13.0 (Adobe Systems Incorporated, San Jose, CA) to quantify the color descriptors of the areas of interest. Color descriptors from digital pictures are a reliable and efficient method to assess nestling mouth coloration (Saino et

al. 2003; Dugas and McGraw 2011; Dugas and Dillow 2013), allowing us to obtain average color estimates of whole areas with minimal manipulation time, thus reducing interference on study subjects. However, this method does not capture ultraviolet reflectance. In pied flycatcher nestlings, the orange palates are bordered by white-yellowish fleshy flanges. To quantify nestling mouth coloration, we recorded the saturation value of the nestling's palate as an indicator of carotenoid pigmentation (Saino et al. 2003; Dugas and McGraw 2011) and the lightness (total reflectance) of the inner and outer parts of the mouth flanges as an indicator of conspicuousness (Kilner and Davies 1998; Wiebe and Slagsvold 2009b). To assess the repeatability of color variables, we measured a subset of 24 individuals twice. The results showed high repeatability for palate saturation (r = 0.69, $F_{22, 25} = 5.41$, $P < 0.001$), inner flange lightness (r = 0.79, $F_{22, 25} = 8.44$, $P < 0.001$), and outer flange lightness (r = 0.89, $F_{22, 25} = 16.97$, $P < 0.001$).

Statistical analyses

We used linear mixed effects models with restricted maximum likelihood to perform statistical analyses (Zuur et al. 2009). We employed the “nlme” package (Pinheiro et al. 2020) in R 4.0.2 (R Development Core Team 2020) to fit the models (See Table S1 for a description of all saturated models). To improve the stability of the models, likelihood of model convergence, and accuracy of parameter estimates (Harrison et al. 2018), we Z-transformed (mean centered with SD of 1) all numerical independent variables. Standardized beta coefficients were used to estimate standardized effect size for comparisons between fixed effects (Schielzeth 2010). In all models, we included original brood size at day 7 and hatching date (1 = June 8th) as covariates among fixed effects because the number of nestmates and differences in resource availability and parental condition as the season progresses could affect adult and nestling behavior. Validation of model assumptions were performed by inspecting residual plots generated by the

“performance” package (Lüdecke et al. 2021) for linear mixed models and the “DHARMa” package for generalized mixed models (Hartig 2022). Predicted model values were plotted using the package “ggeffects” (Lüdecke 2018).

To confirm that the treatment had the intended effect on nestling mass gain, we compared differences in nestling mass between the vitamin E and control groups on days 7 and 9, while controlling for initial mass at day 5 as a covariate. To analyze the effect of treatment on nestling flange conspicuousness, we conducted a principal component analysis (PCA) using the lightness values of the inner and outer areas of the flange with the prcomp() function from the “stats” package. We used individual scores on the first principal component (PC1), which explained 63.2% of the total variance, as a measure of overall lightness, as they were highly correlated with lightness values of both the inner and outer parts of the flange (PC1 loading value = 0.707).

To investigate the effect of vitamin E supplementation on nestling behavior, we built separate models for various dependent variables, including the probability of nestling begging (gaping or not), begging order, postural intensity, profitability of a nestling spatial location, inter-feeding intervals, and the rate of change between nest sectors. We included the sex of the feeding adult entering the nest box as a fixed effect (Wetzel et al. 2020), since nestlings may behave differently according to the parent's sex. To control for intra-brood differences in nestling body mass that could reflect differences in relative size or age, we used the mass difference of each nestling relative to the average of its brood rather than its absolute body mass. The probability of nestling begging was analyzed using a generalized linear mixed model with a binomial error distribution. Although begging order and postural intensity were measured as ordinal variables, they were treated as numeric in linear mixed models. We verified that conclusions did not differ from those from an ordinal regression (Christensen 2022).

Spatial profitability was computed as the fraction of total feedings given by each parent to the sector occupied by each nestling, relative to the parent's location in a given visit. Profitability values were transformed as logits prior to analysis. We also calculated the rate of spatial change between nest sectors for each nestling by dividing the number of changes between sectors by the number of feeding visits. To test whether nestling mobility was related to hunger levels, we computed the mean time of food deprivation for each chick and the average rate of change between sectors per brood by dividing the mean number of position changes by nestlings for the entire brood by the number of feeding visits (McRae et al. 1993). We included the number of caring parents and the degree of overlap between parent sexes in the use of nest sectors as fixed effects in this model to investigate whether the predictability of parental feeding position influenced nestling movement (Kölliker and Richner 2004). We calculated the degree of overlap between parent sexes in the use of nest sectors as the difference between the number of shared nest sectors multiplied by the number of feedings given from these sectors minus the number of feedings given from non-overlapping sectors, divided by the total number of feeding events. This resulted in an index ranging from 0 (no overlap) to 1 (maximum overlap). We assumed that uniparental broods had a maximum (1) overlap.

We analyzed parental feeding preferences using generalized linear mixed models with a binomial error distribution and a binary response variable (1 = fed, 0 = not fed). Since allocation patterns may vary by parental sex (Kilner 2002, Ryser et al. 2016), we conducted separate analyses for females and males. To avoid convergence issues caused by a high number of predictors, we proceeded in two steps. First, we built models with all nestling traits, including relative mass, begging behavior (order and postural intensity), coloration (flange lightness and palate saturation), and spatial profitability, interacting with treatment and hatching date. Second, we built a second group of models considering

only significant predictors from the first step (begging behavior, position and treatment), including all pairwise interactions among them as well as with relative body mass (Table S1). To equalize the predictive potential and precision of spatial profitability with the other predictors, we transformed its raw values into 4-level ranks using the rank() function from the "base" package. We analyzed sex differences in the feeding rate of parents and the duration of each feeding visit to investigate whether differences between females and males in their degree of familiarity with nestlings could explain sex differences in allocation rules (Gottlander 1987; Lucass et al. 2016) (see Table S1).

We explored the prey-testing behavior of parents in three steps. First, we looked for differences between visits where testing occurred and those where it did not. We compared the total number of begging nestlings and the intra-brood coefficient of variation in time of food deprivation, begging order, postural intensity and spatial profitability between visits where testing occurred and those where it did not, including the occurrence of testing (yes or no) as a categorical predictor (Table S1). Second, in a given visit, we compared the behavior of nestlings that were either tested, fed or neither fed nor tested. We built separate models for nestling behavior, relative body mass, and time of food deprivation as response variables. We also included treatment and a new three-level categorical predictor ("Testing") depending on whether a nestling had been tested, fed, or neither fed nor tested in a given visit (Table S1). Third, we investigated whether the probability of a nestling being fed after having been tested varied according to its relative body mass, treatment, and time of food deprivation.

We determined the optimal random- (intercepts and slopes) and fixed-effect structure for each model by comparing nested models with likelihood ratio tests, using a top-down strategy (Zuur et al. 2009) (Table S1). To find out the optimal random structure, we started with the most complex fixed and random structure (Barr et al. 2013) (or no

fixed effects in the case of generalized linear mixed models), and progressively removed random effects one by one, comparing pairs of models with the anova() function from the "stats" package. Next, to determine the optimal fixed structure, we began with the most complex fixed-effect structure and the optimal random structure selected in the previous step. We then progressively removed fixed effects with lower β coefficients one by one, beginning with the interactions, and compared both models by likelihood ratio tests using the anova() function.

Table S1: List of saturated models containing all additive (+) and interaction (x) fixed effects. Predictor variables of interest are shown in bold, with covariates non-bolded. Random effects shown are the optimal random structure selected in each model.

| Response variable | Predictor + covariates | Model type | Random effect * |
|---|---|---|---|
| **NESTLING GROWTH AND MOUTH COLORATION** | | | |
| **Initial body mass day 5** | **Treatment** | LMM | (1\|Nest) |
| **Body mass day 7** | **Treatment** + Mass 5d + Brood size + Date + Treatment × Mass 5d + Treatment × Date | LMM | (Treatment \| Nest) |
| **Body mass day 9** | **Treatment** + Mass 5d + Brood size + Date + Treatment × Mass 5d + Treatment × Date | LMM | (Treatment \| Nest) |
| **Palate saturation** | **Treatment** + Relative mass 7d + Brood size + Date + Treatment × Relative mass 7d + Treatment × Date | LMM | (Treatment \| Nest) |
| **Flange lightness PC1** | **Treatment** + Relative mass 7d + Brood size + Date + Treatment × Relative mass 7d + Treatment × Date | LMM | (1 \| Nest) |
| **NESTLING BEHAVIOR** | | | |
| **Body mass day 7 recorded nestlings** | **Treatment** | LMM | (Treatment \| Nest) |
| **Probability of gaping** | **Treatment** + Deprivation time + Parent sex + Relative mass 7d + Brood size + Date + Treatment × Deprivation time + Treatment × Relative mass 7d + Treatment × Parent sex + Treatment × Date | GLMM | (1 \| Nest/ID) + (0 + Deprivation time \| Nest × ID) + (1 \| Nest × Sequence) |
| **Begging order** | **Treatment** + Deprivation time + Parent sex + Relative mass 7d + Brood size + Date | LMM | (0 + Relative mass 7d \| Nest) + (1 \| Nest/ID) + (1 \| Nest × Sequence) |

| | | | |
|---|---|---|---|
| | + Treatment × Deprivation time + Treatment × Relative mass 7d + Treatment × Parent sex + Treatment × Date | | |
| **Postural intensity** | **Treatment** + Deprivation time + Parent sex + Relative mass 7d + Brood size + Date + Treatment × Deprivation time + Treatment × Relative mass 7d + Treatment × Parent sex + Treatment × Date | LMM | (0 + Relative mass 7d \| Nest) + (0 + Deprivation time \| Nest × ID) + (1 \| Nest/ID) + (1 \| Nest × Sequence) |
| **Position profitability** | **Treatment** + Deprivation time + Parent sex + Relative mass 7d + Brood size + Date + Treatment × Deprivation time + Treatment × Relative mass 7d + Treatment × Parent sex + Treatment × Date | LMM | (0 + Relative mass 7d \| Nest) + (0 + Deprivation time \| Nest × ID) + (1 \| Nest/ID) + (1 \| Nest × Sequence) |
| **Nestling rate of change between nest sectors** | **Treatment** + Average Deprivation time + Parental feeding rate + Relative mass 7d + Brood size + Date + Treatment × Date + Treatment × Average Deprivation time + Treatment × Parental feeding rate + Treatment × Relative mass 7d | LMM | (1 \| Nest) |
| **Average nest rate of change between nest sectors** | Brood size + Date + Number of caring parents + Rate of nest sectors overlap | LM | None |
| **Inter-feeding interval** | **Treatment** + Relative mass 7d + Parent sex + Brood size + Date + Treatment × Relative mass 7d + | LMM | (1 \| Nest) + (1 \| Nest × ID) + (1 \| Nest × Sequence) |

| | | | |
|---|---|---|---|
| | Treatment × Parent sex + Treatment × Date | | |
| **PARENTAL FEEDING BEHAVIOR** | | | |
| **Feeding rate** | Parent sex + Date + Brood size + Parent sex × Date | LM | None |
| **Duration feeding visits** | Parent sex + Date + Brood size + Parent sex × Date | LM | None |
| **Probability of being fed by a male** | **Treatment** + Begging order + Spatial profitability rank + Postural intensity + Relative mass 7d + Palate saturation + Flange lightness PC1 + Brood size + Date + Treatment × Begging order + Treatment × Spatial profitability rank + Treatment × Postural intensity + Treatment × Relative mass 7d + Treatment × Palate saturation + Treatment × Flanges lightness PC1 + Treatment × Date + Begging order × Postural intensity + Begging order × Spatial profitability rank + Begging order × Relative mass 7d + Postural intensity × Spatial profitability rank + Postural intensity × Relative mass 7d + Relative mass 7d × Spatial profitability rank | GLMM | (0 + Begging order + Position profitability rank \| Nest) + (1 \| Nest/ID) + (1 \| Nest × Sequence) |
| **Probability of being fed by a female** | **Treatment** + Begging order + Spatial profitability rank + Postural intensity + Relative mass 7d + Palate saturation + Flange lightness PC1 + Brood size + Date + | GLMM | (0 + Begging order + Position profitability rank \| Nest) + (1 \| Nest/ID) + (1 \| Nest × Sequence) |

| | | | |
|---|---|---|---|
| | Treatment × Begging order + Treatment × Spatial profitability rank + Treatment × Postural intensity + Treatment × Relative mass 7d + Treatment × Palate saturation + Treatment × Flanges lightness PC1 + Treatment × Date + Begging order × Postural intensity + Begging order × Spatial profitability rank + Begging order × Relative mass 7d + Postural intensity × Spatial profitability rank + Postural intensity × Relative mass 7d + Relative mass 7d × Spatial profitability rank | | |
| **PREY-TESTING** | | | |
| **Coefficient of variation of deprivation time** | **Occurrence of testing** + Parent sex + Brood size + Date + Testing × Parent sex + Testing × Date | LMM | (1 \| Nest) |
| **Coefficient of variation of nestlings begging order** | **Occurrence of testing** + Parent sex + Brood size + Date + Testing × Parent sex + Testing × Date | LMM | (1 \| Nest) |
| **Coefficient of variation of postural intensity** | **Occurrence of testing** + Parent sex + Brood size + Date + Testing × Parent sex + Testing × Date | LMM | (1 \| Nest) |
| **Coefficient of variation of position profitability** | **Occurrence of testing** + Parent sex + Brood size + Date + Testing × Parent sex + Testing × Date | LMM | (1 \| Nest) |
| **Number of nestlings** | **Occurrence of testing** + Parent sex + Brood size + | LMM | (1 \| Nest) |

| | | | |
|---|---|---|---|
| | Date + Testing × Parent sex + Testing × Date | | |
| **Begging order** | **Testing**# + **Treatment** + Deprivation time + Parent Sex + Relative mass 7d + Brood size + Date + Testing × Treatment + Testing × Deprivation time + Testing × Parent sex + Testing × Relative mass 7d + Testing × Date | LMM | (1 \| Nest) + (1 \| Nest × ID) + (1 \| Nest × Sequence) |
| **Postural intensity** | **Testing**# + **Treatment** + Deprivation time + Parent Sex + Relative mass 7d + Brood size + Date + Testing × Treatment + Testing × Deprivation time + Testing × Parent sex + Testing × Relative mass 7d + Testing × Date | LMM | (1 \| Nest) + (1 \| Nest × ID) + (1 \| Nest × Sequence) |
| **Position profitability** | **Testing**# + **Treatment** + Deprivation time + Parent Sex + Relative mass 7d + Brood size + Date + Testing × Treatment + Testing × Deprivation time + Testing × Parent sex + Testing × Relative mass 7d + Testing × Date | LMM | (1 \| Nest) + (0+Testing\| Nest × ID) + (1 \| Nest × Sequence) |
| **Relative mass day 7** | **Testing**# + **Treatment** + Deprivation time + Parent Sex + Brood size + Date + Testing × Treatment + Testing × Deprivation time + Testing × Parent sex + Testing × Date | LMM | (1 \| Nest) |
| **Deprivation time** | **Testing**# + **Treatment** + Parent Sex + Relative mass | LMM | (1 \| Nest) + (1 \| Nest × ID) + (1 \| Nest × Sequence) |

| | | | |
|---|---|---|---|
| | 7d + Brood size + Date + Testing × Treatment + Testing × Parent sex + Testing × Relative mass 7d + Testing × Date | | |
| **Probability of being fed after being tested** | **Treatment +** Brood size + Date + Deprivation time + Relative mass 7d + Treatment × Date + Treatment × Deprivation time + Treatment × Relative mass 7d | GLMM | (1 \| Nest) + (1 \| Nest × ID) + (1 \| Nest × Sequence) |

* Optimal random structure selected in each model. Random intercepts account for non-independence between observations of the same level (e.g. nest), and random slopes allow a given predictor to have different effects for each level of the random intercept (nestling, nest or sequence). We included nest ID as a random intercept for models predicting nestling growth and mouth color as response variables, with the effects of treatment as random slope for the nestling growth model and the effects of treatment, relative body mass at day 7, and their interaction as random slopes for the mouth color models. For models predicting nestling behavior (for both feeding and prey-testing by parents), nest ID, nestling ID and sequential visit order (the two latter ones nested within nest) were included as random intercepts. Relative body mass at day 7, treatment and their interaction were also included as random slopes for the random intercept nest ID, and time of food deprivation for the random intercept nestling ID (nested within nest). For models predicting parental feeding and prey-testing as a function of nestling size, behavior, coloration and treatment, we used the same random structure as for models predicting nestling behavior, substituting time of food deprivation by begging order, begging postural intensity and nestling position as random slopes.

# "Testing" is a categorical factor with three levels ("*Tested*", "*Fed*" and "*Neither fed nor tested*" chicks).

References:

Barr DJ, Levy R, Scheepers C, Tily HJ. 2013. Random effects structure for confirmatory hypothesis testing: keep it maximal. J Mem Lang. 68:255-278.

Christensen RHB. 2022. “ordinal—Regression Models for Ordinal Data .” R package version 2022.11-16. https://CRAN.R-project.org/package=ordinal.

Dugas MB, McGraw KJ. 2011. Proximate correlates of carotenoid-based mouth coloration in nestling house sparrows. The Condor. 113:691-700.

Dugas MB, Dillow LL. 2013. Rictal flanges of nestlings birds are most colorful near the gape. Wilson J Ornithol. 125:430-433.

Gómez J, Liñán-Cembrano G. 2017. SpotEgg: an image-processing tool for automatized analysis of colouration and spottiness. J Avian Biol. 48:502-512.

Gottlander K. 1987. Parental feeding behaviour and sibling competition in the pied flycatcher *Ficedula hypoleuca*. Ornis Scand. 18:269-276.

Harrison XA, Donaldson L, Correa-Cano ME, Evans J, Fisher DN, Goodwin CED, Robinson BS, Hodgson DJ, Inger R. 2018. A brief introduction to mixed effects modelling and multi-model inference in ecology. PeerJ. 6:e4794.

Hartig F. 2022. DHARMa: Residual Diagnostics for Hierarchical (Multi-Level / Mixed) Regression Models. R package version 0.4.6, <https://CRAN.R-project.org/package=DHARMa>.

Khayutin SN, Dmitrieva LP, Alexandrov LI. 1988. Psychobiological aspects of the acceleration of postembryonic development in the asynchronous breeder, Pied Flycatcher (*Ficedula hypoleuca*). Int J Comp Psychol. 1:145-166.

Kilner RM. 2002. Sex differences in canary (*Serinus canaria*) provisioning rules. Behav. Ecol. Sociobiol. 52:400-407.

Kilner RM, Davies NB. 1998. Nestling mouth colour: ecological correlates of a begging signal. Anim Behav. 56:705-712.

Kölliker M, Richner H. 2004. Navigation in a cup: chick positioning in great tit, Parus major, nests. Anim Behav. 68:941-948.

Lucass C, Stöwe M, Eens M, Müller W. 2016. Favored parent–offspring trait combinations? On the interplay of parental and offspring traits. Behav Ecol. 27:134-140.

Lüdecke D. 2018. “ggeffects: Tidy Data Frames of Marginal Effects from Regression Models.” J Open Source Softw. 3(26):772. doi:10.21105/joss.00772 <https://doi.org/10.21105/joss.00772>.

Lüdecke D, Ben-Shachar MS, Patil I, Waggoner P, Makowski D. 2021. performance: An R Package for Assessment, Comparison and Testing of Statistical Models. J Open Source Softw. 6(60):3139.

Lundberg A, Alatalo RV. 1992. The pied flycatcher. London, UK: Poyser.

McRae SB, Weatherhead PJ, Montgomerie R. 1993. American robin nestlings compete by jockeying for position. Behav Ecol Sociobiol. 33:101-106.

Pérez-Rodríguez L, Redondo T, Ruiz-Mata R, Camacho C, Moreno-Rueda G, Potti J. 2019. Vitamin E supplementation - but not induced oxidative stress – influences telomere dynamics during early development in wild passerines. Front Ecol Evol. 7: 173.

Pinheiro J, Bates D, DebRoy S, Sarkar D, R Core Team. 2020. nlme: Linear and Nonlinear Mixed Effects Models. R package version 3.1-145, https://CRAN.R-project. org/package=nlme

Porkert J, Špinka M. 2006. Begging in common redstart nestlings: Scramble competition or signalling of need? Ethology. 112:398-410.

R Development Core Team. 2020. R: A language and environment for statistical computing. Vienna,

Austria: R Foundation for Statistical Computing. Retrieved from https://www.R-project. org/

Redondo T, Castro F. 1992. Signaling of nutritional need by magpie nestlings. Ethology. 92:193-204.

Ryser S, Guillod N, Bottini C, Arlettaz R, Jacot A. 2016. Sex-specific food provisioning patterns by parents in the asynchronously hatching European hoopoe. Anim Behav. 117:15-20.

Saino N, Ambrosini R, Martinelli R, Ninni P, Møller AP. 2003. Gape coloration reliably reflects immunocompetence of barn swallow (*Hirundo rustica*) nestlings. Behav Ecol. 14:16-22.

Schielzeth H. 2010. Simple means to improve the interpretability of regression coefficients. Methods Ecol Evol. 1:103-113.

Siikamäki P. 1996. Nestling growth and mortality of Pied Flycatchers *Ficedula hypoleuca* in relation to weather and breeding effort. Ibis. 138:471-478.

Slagsvold T, Wiebe K. 2007. Hatching asynchrony and early nestling mortality: the feeding constraint hypothesis. Anim Behav. 73:691-700.

Wetzel DP, Mutzel A, Wright J, Dingemanse NJ, Ridley A. 2020. Novel sources of (co)variation in nestling begging behavior and hunger at different biological levels of analysis. Behavl Ecol. 31:960-970.

Wiebe KL, Slagsvold T. 2009a. Parental sex differences in food allocation to junior brood members as mediated by prey size. Ethology 115:49-58.

Wiebe KL, Slagsvold T. 2009a. Mouth coloration in nestling birds: increasing detection or signalling quality? Anim Behav. 78:1413-1420.

Wright J, Hinde C, Fazey I, Both C. 2002. Begging signals more than just short-term need: cryptic effects of brood size in the pied flycatcher (*Ficedula hypoleuca*). Behav Ecol Sociobiol. 52:74-83.

Zuur AF, Ieno EN, Walker NJ, Saveliev AA, Smith GM. 2009. Mixed effects models and extensions in ecology with R. New York, USA: Springer.

SUPPLEMENTARY FIGURES AND TABLES

Figure S1: The effect of experimental treatment (V = vitamin E; C = control) on nestling body mass at days 7 (A) and 9 (B) as a function of initial mass at day 5. Shown are raw values and 95% CI (grey bands) for regression lines. The opacity of the points corresponds to the number of overlapping values.

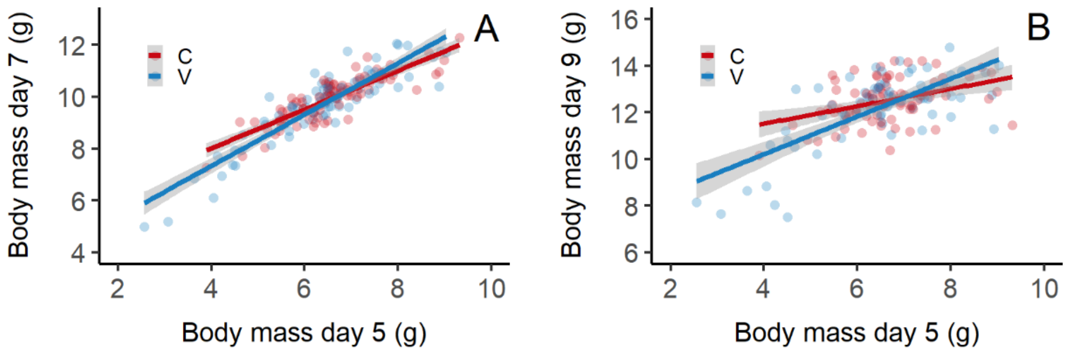

Figure S2: The effect of experimental treatment (V = vitamin E supplementation or C = control) on the relationship between nestling postural intensity and hatching date (in standardized Z values). Shown are predicted model values and their 95% CI (colored bands).

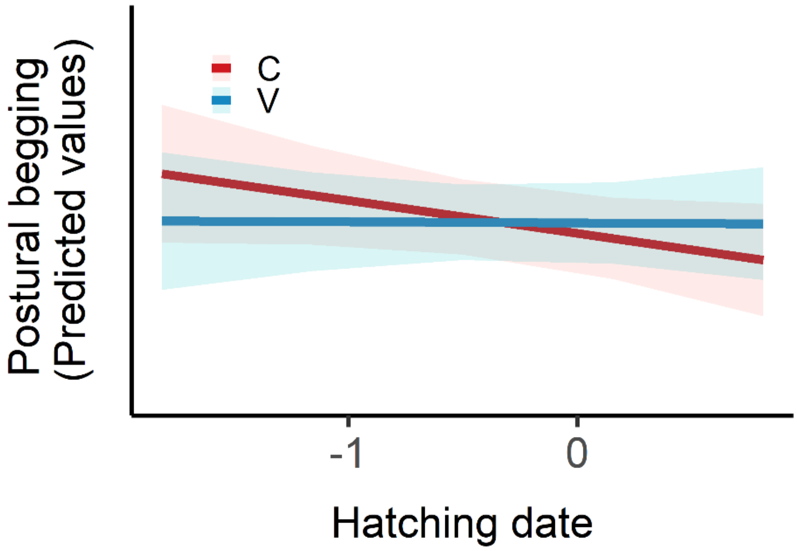

Figure S3: The effect of experimental treatment (V = vitamin E; C = control) and nestling relative body mass at day 7 on the spatial profitability of nestling position. Shown are raw values and 95% CI (grey bands) for regression lines. The opacity of the points corresponds to the number of overlapping values.

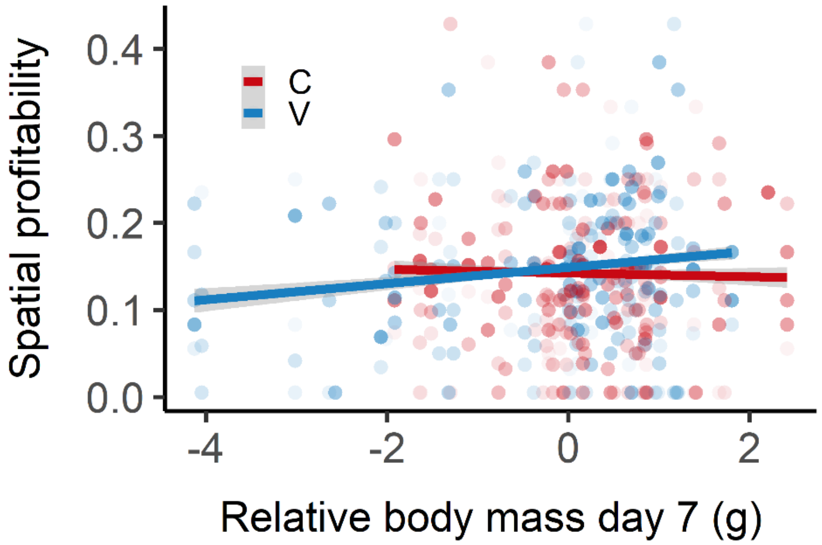

Figure S4: Differences in relative body mass at day 7 between Tested, Fed and Neither fed nor tested nestlings according to the experimental treatment (V = vitamin E; C = control). Shown are means ± 95% CI.

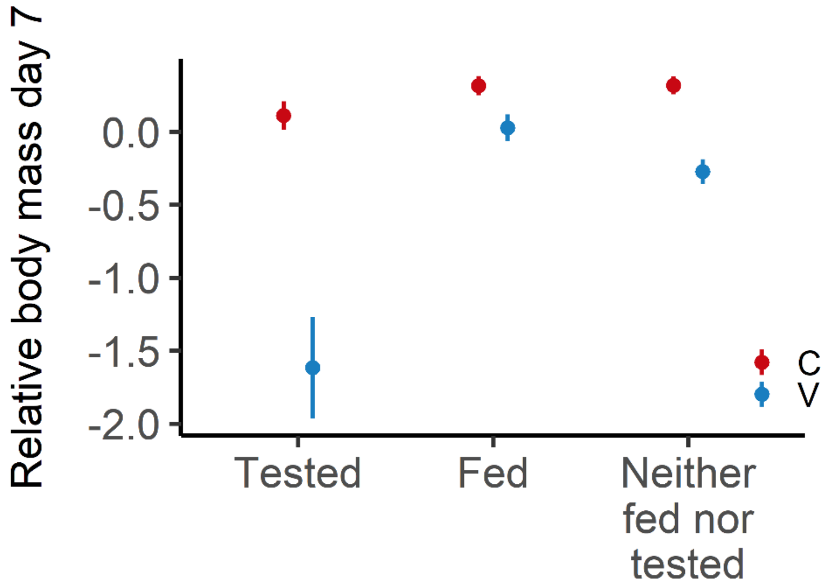

Table S2: Estimated parameters (± SE), and *t* values for models explaining nestling body mass on days 7 and 9. "Treatment" is a categorical variable with "*Control*" as the reference group. Significant *P*-values are indicated in bold.

| | **Estimate** | **SE** | ***t*** | ***P*** |
|---|---|---|---|---|
| **Body mass day 7**[a] | | | | |
| Intercept | 9.871 | 0.061 | 161.5 | **<0.001** |
| Body mass day 5 | 0.964 | 0.056 | 17.301 | **<0.001** |
| Treatment × Body mass day 5 | 0.262 | 0.069 | 3.791 | **<0.001** |
| Brood size | -0.100 | 0.063 | -1.590 | 0.121 |
| Treatment | -0.046 | 0.075 | -0.616 | 0.543 |
| **Body mass day 9**[b] | | | | |
| Intercept | 12.449 | 0.124 | 100.5 | **<0.001** |
| Body mass day 5 | 0.732 | 0.086 | 8.491 | **<0.001** |
| Hatching date | -0.349 | 0.128 | -2.729 | **0.011** |
| Treatment × Body mass day 5 | 0.339 | 0.101 | 3.358 | **0.001** |
| Treatment | -0.130 | 0.107 | -1.214 | 0.235 |

[a] Predictors (hatching date) and their interactions (Supplementary Table S1) that failed to improve model fit according to a likelihood ratio test are not depicted.

[b] Predictors (brood size) and their interactions (Supplementary Table S1) that failed to improve model fit according to a likelihood ratio test are not depicted.

Table S3: Estimated parameters (± SE), and *t* values for models explaining nestling mouth coloration (palate saturation and flange lightness PC1). "Treatment" is a categorical variable with "*Control*" as the reference group. Significant *P*-values are indicated in bold.

| | **Estimate** | **SE** | ***t*** | ***P*** |
|---|---|---|---|---|
| **Palate saturation**[a] | | | | |
| Intercept | 0.803 | 0.006 | 139.5 | **<0.001** |
| Hatching date | 0.012 | 0.005 | 2.624 | **0.014** |
| Body mass | 0.003 | 0.002 | 1.697 | 0.092 |
| Treatment | -0.000 | 0.005 | -0.079 | 0.938 |
| **Flange lightness PC1**[b] | | | | |
| Intercept | -0.041 | 0.152 | -0.271 | 0.788 |
| Treatment | 0.069 | 0.146 | 0.474 | 0.636 |

[a] Predictors (brood size) and their interactions (Supplementary Table S1) that failed to improve model fit according to a likelihood ratio test are not depicted.

[b] Predictors (body mass, brood size and hatching date) and their interactions (Supplementary Table S1) that failed to improve model fit according to a likelihood ratio test are not depicted.

Table S4: Estimated parameters (± SE), and *t* values for models explaining nestling inter-feeding intervals. "Treatment" is a categorical variable with "*Control*" as the reference group. Significant *P*-values are indicated in bold.

| | **Estimate** | **SE** | ***t*** | ***P*** |
|---|---|---|---|---|
| Intercept | 72.47 | 0.951 | 76.19 | **<0.001** |
| Body mass | -1.723 | 0.549 | -3.141 | **0.002** |
| Treatment | -0.492 | 1.082 | -0455 | 0.651 |

Predictors (parent sex, brood size and hatching date) and their interactions (Supplementary Table S1) that failed to improve model fit according to a likelihood ratio test are not depicted.

Table S5: Estimated parameters (± SE), and *t* values for models explaining average (brood) nest rate of change between nest sectors. "Treatment" is a categorical variable with "*Control*" as the reference group. Significant *P*-values are indicated in bold.

| | **Estimate** | **SE** | ***t*** | ***P*** |
|---|---|---|---|---|
| Intercept | 0.387 | 0.052 | 7.377 | **<0.001** |
| Index of parental overlap | -0.044 | 0.035 | -1.264 | 0.219 |
| Brood size | 0.034 | 0.028 | 1.207 | 0.240 |
| Number of caring parents | -0.033 | 0.070 | -0.473 | 0.641 |
| Hatching date | -0.003 | 0.028 | -0.125 | 0.901 |

Interactions between predictors (Supplementary Table S1) that failed to improve model fit according to a likelihood ratio test are not depicted.

Table S6: Estimated parameters (± SE), and *t* values for models explaining differences in number of nestlings and the intra-brood coefficients of variation in the time of food deprivation, begging order, postural intensity and spatial profitability between sequences with and without testing events (both sexes combined). "Occurrence of testing" is a categorical variable with two levels and "*No occurrence of testing*" as the reference group. Significant *P*-values are indicated in bold.

| | **Estimate** | **SE** | ***t*** | ***P*** |
|---|---|---|---|---|
| **Number of nestlings begging**[a] | | | | |
| Intercept | 2.430 | 0.086 | 28.32 | **<0.001** |
| Occurrence of testing | 0.549 | 0.102 | 5.364 | **<0.001** |
| **Deprivation time (CV)**[b] | | | | |
| Intercept | -2.096 | 0.081 | -25.98 | **<0.001** |
| Occurrence of testing × Hatching date | 0.464 | 0.171 | 2.711 | **0.007** |
| Occurrence of testing | -0.444 | 0.165 | -2.693 | **0.007** |
| Hatching date | -0.062 | 0.083 | -0.744 | 0.463 |
| **Begging order (CV)**[c] | | | | |
| Intercept | 3.809 | 0.031 | 124.5 | **<0.001** |
| Brood size | 0.055 | 0.028 | 1.959 | 0.065 |
| Occurrence of testing | 0.115 | 0.061 | 1.891 | 0.059 |
| **Postural intensity (CV)**[d] | | | | |
| Intercept | 2.280 | 0.136 | 16.81 | **<0.001** |
| Brood size | 0.320 | 0.148 | 2.160 | **0.042** |
| Occurrence of testing | -0.367 | 0.244 | -1.504 | 0.148 |
| **Spatial profitability (CV)**[e] | | | | |
| Intercept | 3.627 | 0.121 | 30.08 | **<0.001** |
| Occurrence of testing | 0.035 | 0.106 | 0.328 | 0.743 |

[a] Predictors (parent sex, brood size and hatching date) and their interactions (Supplementary Table S1) that failed to improve model fit according to a likelihood ratio test are not depicted.

[b] Predictors (parent sex and brood size) and their interactions (Supplementary Table S1) that failed to improve model fit according to a likelihood ratio test are not depicted.

[c] Predictors (parent sex and hatching date) and their interactions (Supplementary Table S1) that failed to improve model fit according to a likelihood ratio test are not depicted.

[d] Predictors (parent sex and hatching date) and their interactions (Supplementary Table S1) that failed to improve model fit according to a likelihood ratio test are not depicted.

[e] Predictors (parent sex, brood size and hatching date) and their interactions (Supplementary Table S1) that failed to improve model fit according to a likelihood ratio test are not depicted.
